# Epidemiology of La Crosse Virus Emergence, Appalachia Region, United States

**DOI:** 10.3201/eid2211.160308

**Published:** 2016-11

**Authors:** Sharon Bewick, Folashade Agusto, Justin M. Calabrese, Ephantus J. Muturi, William F. Fagan

**Affiliations:** University of Maryland, College Park, Maryland, USA (S. Bewick, W.F. Fagan);; University of Kansas, Lawrence, Kansas, USA (F. Agusto); Smithsonian Conservation Biology Institute, Front Royal, Virginia, USA (J.M. Calabrese);; Illinois Natural History Survey, Champaign, Illinois, USA (E.J. Muturi)

**Keywords:** La Crosse virus, Bunyaviridae, eastern tree-hole mosquito, Ochlerotatus triseriatus, Asian tiger mosquito, Aedes albopictus, compartmental model, invasive vector, viruses, vector-borne infections, Appalachia, United States

## Abstract

La Crosse encephalitis is a viral disease that has emerged in new locations across the Appalachian region of the United States. Conventional wisdom suggests that ongoing emergence of La Crosse virus (LACV) could stem from the invasive Asian tiger (*Aedes albopictus*) mosquito. Efforts to prove this, however, are complicated by the numerous transmission routes and species interactions involved in LACV dynamics. To analyze LACV transmission by Asian tiger mosquitoes, we constructed epidemiologic models. These models accurately predict empirical infection rates. They do not, however, support the hypothesis that Asian tiger mosquitoes are responsible for the recent emergence of LACV at new foci. Consequently, we conclude that other factors, including different invasive mosquitoes, changes in climate variables, or changes in wildlife densities, should be considered as alternative explanations for recent increases in La Crosse encephalitis.

## Abstract 2

Emergence may involve invasive mosquitoes other than Asian tiger mosquitoes, climate change, and changes in wildlife densities.

In recent years, several vectorborne diseases have reemerged either at new locations or to new levels in locations where they have historically ranged. Commonly cited factors for reemergence include evolution of novel vector or pathogen strains (*1*), increased human mobility or disease spread by infected travelers, decreased herd immunity (*2*), landscape change (*3*), climate change (*4*), and invasion of new regions by competent disease vectors (*5*). Although disease translocations across continents are almost always a result of human transport, pathogens that exhibit novel regional spread, increased transmission in preexisting locations, or both are more difficult to explain. Such is the case with La Crosse encephalitis, a mosquitoborne viral disease currently emerging in the US Appalachian region (Appalachia, comprising Tennessee, North Carolina, Virginia, and West Virginia). With 30–180 cases of severe LACV disease reported annually (*6*) and an estimated total disease annual incidence as high as 300,000 cases, LACV is rapidly becoming a leading cause of encephalitis in the United States (*7*,*8*). For patients with severe cases, LACV disease has lifelong neurologic consequences (*6*) and carries an estimated fatality rate of 0.5%–1.9% (*6*,*9*).

Previously, most LACV disease cases were associated with forested areas in the midwestern United States (*10*), where LACV was maintained through a cycle involving the eastern tree-hole mosquito (*Ochlerotatus triseriatus*), hereafter called the tree-hole mosquito, and mammals of 3 species: eastern chipmunks (*Tamias striatus*), gray squirrels (*Sciurus carolinensis*), and fox squirrels (*Sciurus niger*) (*10*,*11*). However, since the mid-1990s, Appalachia has emerged as a new focus for the disease (*8*,*12*–*14*). One potential explanation is the introduction of the invasive Asian tiger mosquito (*Aedes albopictus*), hereafter called the tiger mosquito (*15*). This suggestion is based on the laboratory-demonstrated competence of the tiger mosquito (*16*,*17*), isolation of LACV from field-collected tiger mosquito pools (*18*), observation of LACV-positive tiger mosquitoes at sites of LACV infections of humans (*19*), and the coincidental link between tiger mosquito invasion and the emergence of LACV in the Appalachian region (*12*). Unfortunately, although these observations demonstrate the potential for the tiger mosquito to influence LACV dynamics, the contribution of this mosquito to observed increases in LACV transmission remains unclear. One obstacle to identifying the role of the tiger mosquito in LACV emergence is our limited understanding of the interaction between invasive species and native disease cycles and how this interaction affects disease transmission, both within natural reservoirs and to human hosts. Epidemiologic modeling is a powerful tool, useful for understanding the outcomes of different transmission pathways in other disease systems. To our knowledge, however, no dynamic models for LACV disease have been developed, even for regions where the tree-hole mosquito is the only disease vector. We therefore developed a series of compartmental models (Figure 1) for LACV. Using these models, we then explored LACV dynamics in systems with (native) and without (invaded) tiger mosquitoes to assess the likelihood that the tiger mosquito is responsible for the emergence of LACV in Appalachia.

**Figure 1:**
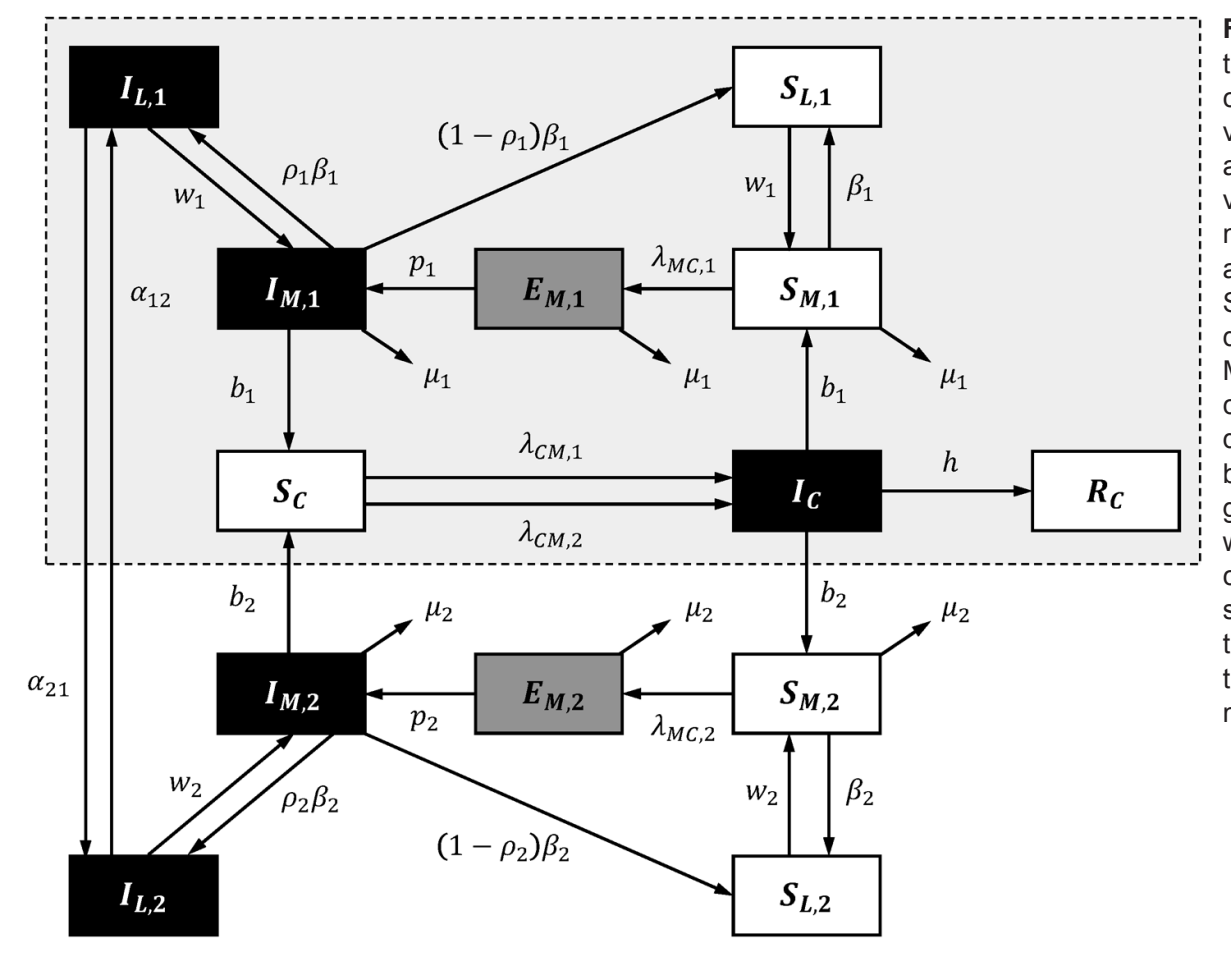
Schematic illustrating transitions/interactions in the compartmental model for La Crosse virus disease. Subscripted 1, 2, and C denote parameters and state variables for the eastern tree-hole mosquito, Asian tiger mosquito, and host populations, respectively. State variables and parameters are described elsewhere (see Basic Model and Table A1, at http://www.clfs.umd.edu/biology/faganlab/disease-ecology.html). Black boxes indicate infected classes; gray boxes, exposed classes; and white boxes, susceptible/recovered classes. Dashed box with gray shading demarks the subset of transitions/interactions that define the native system before tiger mosquito invasion.

## Methods

### Model

We built 3 models: 1) the tree-hole model, in which the tree-hole mosquito is the only LACV vector; 2) the tiger model, in which the tiger mosquito is the only LACV vector; and 3) the tree-hole/tiger model, in which mosquitoes of both species simultaneously serve as LACV vectors. In the third model, mosquitoes of either species may be driven extinct through competitive exclusion; thus, although both vectors are potentially present, it is possible that only 1 persists. For all models, we assumed that the vertebrate host was the eastern chipmunk. The basic dynamic system (Figure 1), including all relevant assumptions and system parameterizations, is fully described elsewhere (see Basic Model, at http://www.clfs.umd.edu/biology/faganlab/disease-ecology.html).

### Basic Reproduction Number, *R*_0_

To determine whether sustained LACV transmission is predicted, we considered the basic reproduction number, *R*_0_, for each of the 3 models (see Basic Reproduction Numbers, *R*_0_, at http://www.clfs.umd.edu/biology/faganlab/disease-ecology.html). For *R*_0_*>1*, LACV transmission can be sustained. For *R*_0_*<1*, LACV will go extinct.

### Transmission Pathways

The tree-hole/tiger model, which represents current LACV spread throughout much of Appalachia and the Midwest, accounts for 2 vector species and, thus, 4 transmission pathways: 1) horizontal transmission between tree-hole mosquitoes and chipmunks, 2) vertical (transovarial) transmission by tree-hole mosquitoes, 3) horizontal transmission between tiger mosquitoes and chipmunks, and 4) vertical transmission by tiger mosquitoes. To determine the relative contributions of the different transmission pathways to *R*_0_, we used elasticity analyses (*20*). Large elasticities indicate transmission routes that contribute most to disease maintenance and spread (see Elasticity Analysis of Transmission Pathways, at http://www.clfs.umd.edu/biology/faganlab/disease-ecology.html).

### LAC Dynamics

*R*_0_ analyses reflect equilibrium conditions, which are good approximations of full system behavior when seasonality is weak or when the system reaches equilibrium rapidly within a single season. Because the relevance of seasonality for LACV is unknown, we also considered fully dynamic multiseason extensions to each of our models (see LAC Dynamics, at http://www.clfs.umd.edu/biology/faganlab/disease-ecology.html). Using dynamic simulations, we estimated the fraction of scenarios (i.e., parameter combinations) resulting in sustained LACV transmission above a critical threshold. This is the numerical equivalent of *R*_0_ but may differ as a result of seasonality. For dynamic simulations in which LACV persists, we also quantified season-long host seroprevalence rates, peak rates of mosquito infection, and the timing of peak transmission to humans. Last, we considered the potential for the tiger mosquito to act as a bridge vector, linking LACV transmission in wildlife reservoirs to infections in human populations.

### Latin Hypercube Sampling

Measurements of system parameters vary, for example, as a result of geographic differences in abiotic variables, differences in local mosquito or chipmunk populations, differences in circulating virus strains, or measurement error. To capture model predictions over empirically determined parameter ranges, we used Latin hypercube sampling, followed by sensitivity analyses with partial rank correlation coefficients (PRCCs) (see Latin Hypercube Sampling and PRCC, at http://www.clfs.umd.edu/biology/faganlab/disease-ecology.html) (*21*).

## Results

### Basic Reproduction Number, *R*_0_

**Figure 2:**
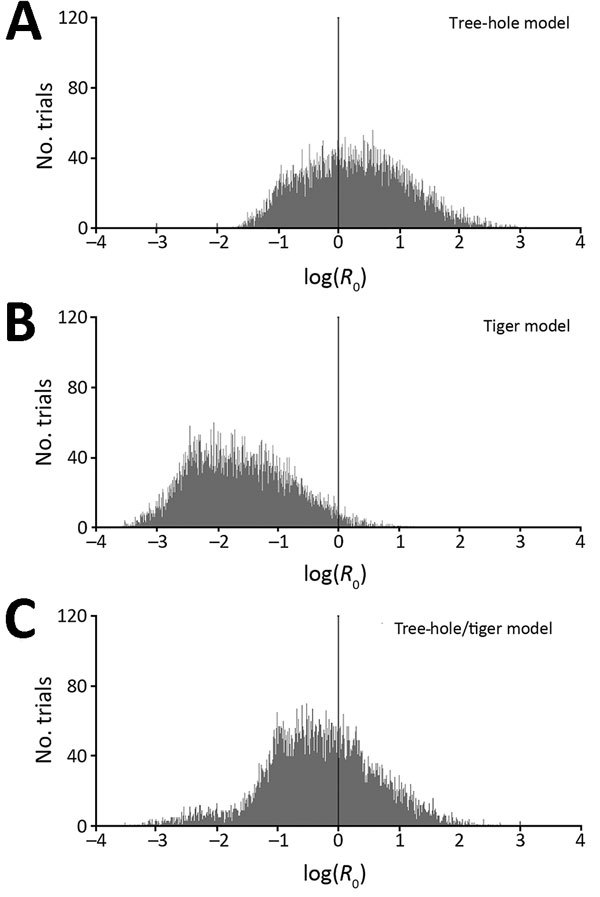
Histograms of basic reproduction numbers (*R*_0_) for La Crosse virus, based on Latin hypercube sampling analyses with 10,000 randomly selected parameter sets (ranges shown at http://www.clfs.umd.edu/biology/faganlab/disease-ecology.html). A) Tree-hole model, B) tiger model, and C) tree-hole and tiger model. In each panel, the black vertical line at log(*R*_0_) = 0 corresponds to the general breakpoint between growing and shrinking infection rates and thus represents the threshold for La Crosse virus disease persistence.

We found that sustained LACV transmission can occur according to most (60%) tree-hole model scenarios but only a small fraction (3%) of tiger model scenarios (Figure 2) (see Latin Hypercube Sampling and PRCC, and Tables A2 A3, at http://www.clfs.umd.edu/biology/faganlab/disease-ecology.html). This finding is surprising because the average tiger mosquito population has approximately twice as many biting females per hectare as does the average tree-hole mosquito population (see Table A2, at http://www.clfs.umd.edu/biology/faganlab/disease-ecology.html), which reflects the higher larval carrying capacity and faster larval maturation rate of tiger mosquitoes than those of tree-hole mosquitoes. Clearly, the numerical abundance of tiger mosquitoes does not compensate for the lower rates of horizontal and vertical LACV transmission and the lower rates of their biting on key host species (see Basic Model, at http://www.clfs.umd.edu/biology/faganlab/disease-ecology.html).

In the 2-vector system, our results for the tree-hole/tiger model indicated a similar outcome—that the invasion of tiger mosquitoes into tree-hole mosquito populations should reduce the fraction of scenarios (from 60% to 37%) in which LACV transmission is viable. Thus, instead of causing the emergence of new LACV foci, tiger mosquitoes should instead drive LACV out of regions where previously it could persist. This result is again a function of the poor intrinsic capability of tiger mosquitoes to serve as LACV vectors. It also depends on asymmetric competition between tiger and tree-hole mosquitoes (see Latin Hypercube Sampling and PRCC and Table A3, at http://www.clfs.umd.edu/biology/faganlab/disease-ecology.html). For example, whereas 14% of parameter combinations yielded tiger mosquitoes competitively excluding tree-hole mosquitoes, for only 0.03% of parameter combinations was the converse true. Moreover, even when tiger and tree-hole mosquitoes were predicted to coexist, the tree-hole mosquito population declined by an average of 63% through interspecific competition. By contrast, interspecific competition reduced the tiger mosquito population by an average of only 16%. Not surprisingly, then, when both mosquito species were present, most (average 78%) were tiger mosquitoes. Because the tiger mosquito is the less competent of the 2 LACV vectors, its invasion actually reduces the likelihood of LACV transmission.

### Transmission Pathways

**Figure 3:**
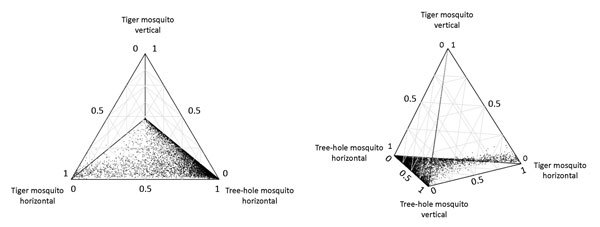
Two views of a quaternary plot showing the relative contributions to basic reproduction number (*R*_0_) from 1) horizontal transmission of La Crosse virus by eastern tree-hole mosquitoes, 2) vertical transmission by eastern tree-hole mosquitoes, 3) horizontal transmission by Asian tiger mosquitoes, and 4) vertical transmission by Asian tiger mosquitoes. This figure plots only the 8,602 replicates (out of 10,000) wherein Asian tiger and eastern tree-hole mosquitoes coexisted. Parameters for each replicate were sampled from the ranges according to our Latin hypercube sampling scheme (http://www.clfs.umd.edu/biology/faganlab/disease-ecology.html).

Elasticity analysis of the 4 virus transmission pathways in the tree-hole/tiger model indicated that in most scenarios the pathway that contributes most to disease spread is horizontal transmission by tree-hole mosquitoes (Figure 3). Vertical transmission by tree-hole mosquitoes can also contribute but usually only when the role of tiger mosquitoes is minimal. For scenarios in which tiger mosquitoes contribute to spread, the main pathway is horizontal transmission either by tiger mosquitoes alone or in combination with horizontal transmission by tree-hole mosquitoes. By contrast, vertical transmission by tiger mosquitoes rarely affects disease dynamics; when it does, it is only in systems in which horizontal transmission by tiger mosquitoes is already the major mode of disease spread.

### LACV Dynamics

Predictions from dynamic models were similar to predictions for *R*_0_ (LACV transmission in 46% of scenarios) and matched many expectations from LACV systems (Table; see also LAC Dynamics at http://www.clfs.umd.edu/biology/faganlab/disease-ecology.html). First, in the native system (the tree-hole model) and the invaded system (the tree-hole/tiger model), predicted host seroprevalence rates were remarkably high, approaching 100% toward the end of the season (mean [median] end-of-season host seroprevalence rates of 89% [99%] in the tree-hole model and 84% [97%] in the tree-hole/tiger model). These rates are consistent with findings from Wisconsin where, at least in high-quality mosquito habitats, multiple surveys have demonstrated that antibody prevalence rates among chipmunks can be well over 50%, often nearing 100% late in the season (*15*,*22*). At the same time, our model predicted very low numbers of LACV-positive mosquitoes, even in the native system (mean [median] yearly averages of 2.0% [1.6%] for the tree-hole model). Again, this finding is highly consistent with observed minimum field infection rates that range from 0.26 to 12.5 (*14*,*23*–*26*). Of note, predicted rates of infection among overwintering eggs were even lower than rates of infection among adult populations (mean [median] end-of-season infection rates of 0.63% [0.49%] for the tree-hole model.) This finding reflects the fact that transovarial transmission is <100% and that overwintering eggs are laid later in the season, sometimes after peak LACV transmission has subsided. Again, predicted rates of egg infection strongly agree with field data indicating that 0.29%–0.6% of overwintering eggs from LACV-endemic sites yield LACV-positive larvae (*26*,*27*). Last, our predicted timing of peak risk for human disease was broadly consistent with observed cases of LACV disease in humans that tend to occur in late summer and early fall (*8*, *13*).

What our dynamic model did not predict was any increase in LACV prevalence in the invaded system (tree-hole/tiger model) over that in the native system (tree-hole model). Even in systems in which LACV survived introduction of the tiger mosquito, the tiger mosquito tended to decrease LACV transmission. For example, both the absolute number of infected mosquitoes and the rate of mosquito infection were lower in the tree-hole/tiger model than in the tree-hole model (Table). Consistent with rates of mosquito infection, we found that rates of host seroprevalence were also lower when tiger mosquitoes were present.

**Table:** Summary statistics for epidemiologic metrics of LACV, based on Latin hypercube sampling analysis of the full dynamic model*

Variable	Tree-hole model	Tiger model	Tree-hole/tiger model†
Parameter sets with LACV persistence, %	46	0.20	24
End-of-season host seroprevalence rate, %			
Mean	89	79	84
Median	99	88	97
Maximum	100	100	100
Midseason host seroprevalence rate, %			
Mean	65	12	18
Median	74	8.9	12
Maximum	100	38	98
Peak no. infected mosquitoes, per hectare			
Mean	32	58	23
Median	22	50	16
Maximum	331	200	222
Peak mosquito infection rate, %			
Mean	4.5	1.6	1.9
Median	3.5	1.5	1.3
Maximum	27	5.3	15
Average mosquito infection rate, %			
Mean	2.0	0.44	0.80
Median	1.6	0.33	0.57
Maximum	13	1.8	6.8
Maximum human transmission, infections per month per person per hectare			
Mean	15	59	14
Median	8.6	40	7.9
Maximum	251	247	221
Timing of peak human transmission			
Mean	Aug 14	Sep 21	Aug 23
Median	Aug 10	Sep 28	Aug 21
Earliest	Jun 21	Aug 26	Jun 26
Latest	Sep 30‡	Sep 30‡	Sep 30‡
End-of-season egg infection rates, %			
Mean	0.63	0.08	0.28
Median	0.49	0.07	0.20
Maximum	5.0	0.32	2.2

*All metrics beyond the first row are only calculated for the subset of simulations that gave infected mosquitoes. LAVC, La Crosse virus. †We avoid reporting minimum values since these are likely to depend on the threshold that we selected for determining disease persistence (see LAC Dynamics, at http://www.clfs.umd.edu/biology/faganlab/disease-ecology.html). ‡In these systems, the abundance of infected mosquitoes was still increasing at the end of the season, indicating that infection rates do not slow before the decline in mosquitoes at the end of the summer.

Although the tiger mosquito is a poor amplifying vector for LACV, it may still increase the number of human LACV infections. Indeed, because this species is an aggressive human biter, it has the potential to intensify the rate of disease transfer to human populations, albeit while simultaneously reducing disease spread in wildlife reservoirs (i.e., it may act as a bridge vector). However, this potential is not realized (Table). Although rates at which tiger mosquitoes bite humans (see Basic Model, at http://www.clfs.umd.edu/biology/faganlab/disease-ecology.html) partially compensated for lower rates of LACV transmission among wildlife reservoirs, this compensation was not complete. Thus, human infections were still predicted to occur more commonly in the native system (Table; see also Summary Statistics for Alternate Scenarios at http://www.clfs.umd.edu/biology/faganlab/disease-ecology.html).

### Sensitivity Analysis with Partial Rank Correlation Coefficients

**Figure 4:**
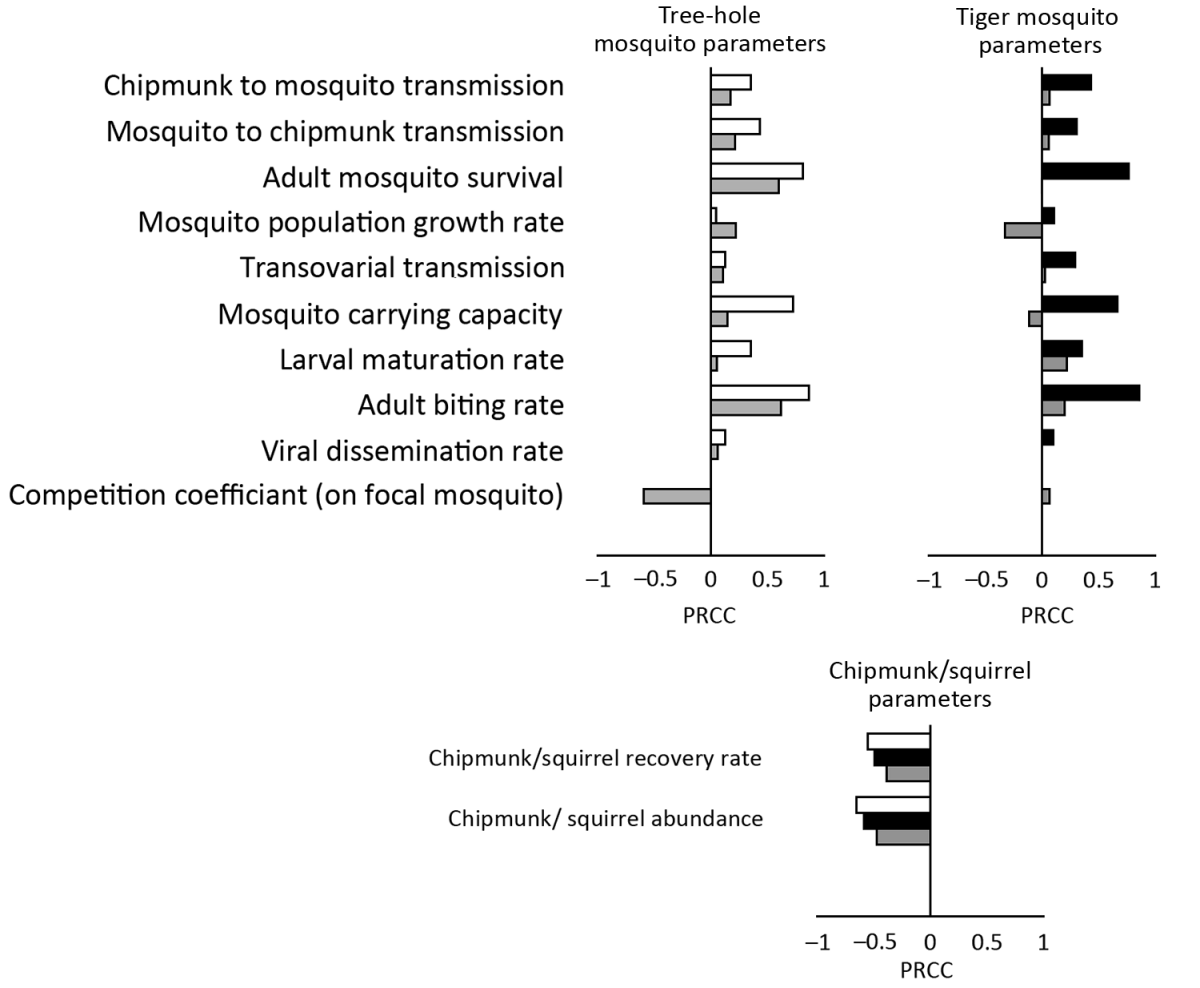
Partial rank correlation coefficients (PRCCs) showing the effect of each model parameter of La Crosse virus transmission on the basic reproduction number (*R*_0_) in the tree-hole model (white), tiger model (black), and tree-hole/tiger model (gray). Positive PRCC values indicate that *R*_0_ is positively correlated with a specific parameter, whereas negative PRCC values indicate the opposite. For specific PRCC values and significance, see Latin Hypercube Sampling and PRCC, at http://www.clfs.umd.edu/biology/faganlab/disease-ecology.html.

In 1-vector models, positive correlation with *R*_0_ was found for transmission rates, biting rates, mosquito survival rates, mosquito population growth rates, mosquito maturation rates, mosquito carrying capacity, and rates of LACV dissemination among mosquitoes (Figure 4). In contrast, rates of host recovery were negatively correlated with *R*_0_, as was host population size. Although this latter result is somewhat counterintuitive, it is well known for systems with a saturating functional response (*28*).

In the 2-vector model, most PRCC values were reduced but maintained the same sign. This finding reflects the similar effect but lesser role of either mosquito species individually when both species are present. Not surprisingly, PRCC reductions were more severe for the tiger mosquito, which is the less competent vector in the 2-vector system. Although most PRCC values merely exhibited reductions in the 2-vector model, several underwent more striking changes. First, the tiger mosquito population growth rate and the tiger mosquito carrying capacity switched from being positively correlated with *R*_0_ (strongly so in the case of carrying capacity) in the tiger model to being negatively correlated with *R*_0_ in the tree-hole/tiger model. This switch occurs because tiger mosquitoes are generally detrimental to LACV spread in systems in which the native vector is also present, a conclusion that accords with our general finding that tiger mosquitoes should, if anything, reduce LACV transmission. Second, the population growth rate of the tree-hole mosquito actually became more strongly correlated with *R*_0_ when tiger mosquitoes were present. The explanation is as follows. In the tree-hole/tiger model, this parameter helps to influence the outcome of interspecific competition. Specifically, high tree-hole mosquito growth rates give this species an increased chance for survival against the more aggressive, generally more fecund, tiger mosquito population.

Although PRCC analyses can identify correlations between model parameters and disease outcomes, large PRCC values additionally indicate model parameters that contribute a high degree of uncertainty to model predictions. In the tree-hole/tiger model, the largest sources of uncertainty in *R*_0_ were the survival rate of tree-hole mosquitoes, the biting rate of tree-hole mosquitoes, and interspecific competition of tiger and tree-hole mosquitoes. In the tree-hole model and the tiger model, the largest contributions to uncertainty were survival and biting rates but also vector carrying capacities.

## Discussion

In contrast to previously published conclusions (*29*), our model suggests that LACV should be sustainable in 46%–60% of scenarios in which the tree-hole mosquito serves as the sole vector. This conclusion still indicates a sizeable number of scenarios in which LACV transmission should not occur. One interpretation is that LACV spread is only marginally favorable and that small changes in system characteristics (e.g., different mosquito or virus strains or environmental conditions) are sufficient to initiate or suppress disease transmission. This marginal favorability could explain the patchy detection of LACV across its native range (*9*) and the sudden appearance of LACV at sites where it was previously absent. The potential for variability in epidemiologic parameters raises the question of how to predict when and where LACV might emerge. Although measuring every system parameter at every local site is not feasible, our PRCC analysis suggests that careful attention to vector survival, competition, biting rates, and carrying capacities would be beneficial.

One factor that does not explain the emergence of novel LACV foci is the invasion of tiger mosquitoes. We predict that the invasive tiger mosquito should actually reduce disease transmission in wildlife reservoirs and human populations (even accounting for the fact that tiger mosquitoes are aggressive human biters). Thus, the presence of the invasive tiger mosquito does not sufficiently explain the dramatic increase in LACV disease cases in Appalachia (*8*,*12*–*14*,*30*) (http://trace.tennessee.edu/utk_gradthes/1788/), suggesting that correlations between tiger mosquito invasion and the epidemiologic risk for LACV disease are driven by other, concomitant, changes. In support of this conclusion is the absence of any increase in LACV disease prevalence in the Midwest, despite the presence of a tiger mosquito infestation. Indeed, reported cases in the region have decreased (*12*), consistent with predictions from our model, but may also be independent of the arrival of tiger mosquitoes (see Midwest LAC Cases, at http://www.clfs.umd.edu/biology/faganlab/disease-ecology.html).

Having ruled out a straightforward contribution of invading tiger mosquitoes to LACV disease emergence, we consider the possibility that tiger mosquitoes are responsible for recent changes in LACV epidemiology. One potential mechanism involves indirect effects on the native vector. Tree-hole mosquitoes that survive competition with tiger mosquitoes are generally larger and more competent LACV vectors (*31*), which could increase the likelihood of LACV transmission. Another possible mechanism is niche differentiation. In general, mosquito competition is quantified by raising the larvae of competing species together in 1 container and then assessing growth metrics such as survival or maturation time. Although this approach enables estimation of direct competition, it does not capture spatial (*32*) or temporal niche partitioning that can decrease the strength of interspecific competition. If tiger mosquitoes do not reduce the tree-hole population as severely as our model predicts, then their dampening effect on LACV transmission will, likewise, be diminished (see Conditions Under Which Tiger Mosquitoes Enable LAC Spread at http://www.clfs.umd.edu/biology/faganlab/disease-ecology.html). Last, our estimates for LACV transmission to and from tiger mosquitoes are based on 1 study that used an LACV strain predating establishment of the tiger mosquito in the United States (*17*). Given that transmission studies can be highly variable and that, since introduction of the tiger mosquito into the United States, local LACV strains may have adapted to be more suitable in this new host, it is also possible that our estimates for tiger mosquito competence are overly low (see Conditions Under Which Tiger Mosquitoes Enable LAC Spread, at http://www.clfs.umd.edu/biology/faganlab/disease-ecology.html). Recent evidence finding substantial infection rates in a tiger mosquito population in Tennessee (*33*) supports this conclusion, although further transmission studies and analyses of virus evolution are warranted.

One final explanation for the recent emergence of LACV, and the explanation that we favor, is that our model predictions are correct and that other factors beyond tiger mosquitoes are responsible for the change. A promising contender is the Asian bush mosquito (*Ochlerotatus japonicus*), hereafter referred to as the bush mosquito. This mosquito is a second container-breeding invasive species that, like the tiger mosquito, seems to have arrived in North America in a shipment of tires (*34*). Because the bush mosquito was introduced more recently than the tiger mosquito (*34*), it has not been studied as extensively, particularly in the context of LACV. Nevertheless, laboratory work has demonstrated its competence as an LACV vector (*35*), and LACV has been isolated from field-collected pools of these mosquitoes (*36*). The role of bush mosquitoes in LACV transmission may be studied by using a model similar to that presented here. However, this study would require additional empirical work, including characterization of transovarial transmission by this species.

Beyond the introduction of novel vectors, other changes (e.g., climatologic variables [*4*], human demographics [*37*], wildlife densities [*38*], and land use [https://vtechworks.lib.vt.edu/handle/10919/64932]) may also contribute to LACV emergence. According to our PRCC analysis, for example, small changes in adult mosquito survival rates could dramatically alter *R*_0_. Mosquito survival rates not only increase the equilibrium size of mosquito populations but also increase the likelihood of mosquitoes surviving to their second or third blood meals, which is necessary for horizontal LACV transmission. Decreases in mosquito predators, varying from birds to spiders (*38**–**41*), could thus strongly affect LACV prevalence. Our PRCC analysis also indicates that mosquito carrying capacities have a substantial effect on LACV persistence. Consequently, even small increases in container availability (e.g., new tire yards or unemptied backyard planters) should have dramatic effects on LACV disease incidence rates. Last, substantial growth has occurred in southern Appalachia over the past 30 years (*37*); thus, even without increased enzootic transmission, absolute cases may have increased from population growth alone. Although purely speculative, such habitat and demographic changes may be the true cause of the recent emergence of LACV.

That tiger mosquito invasion is not predicted to increase LACV transmission or even human cases highlights an important issue at the interface between disease ecology and invasion biology. In particular, this finding shows that predicting whether an invasive vector will exacerbate or dampen the spread of a disease can be complex and can depend on an elaborate network of species interactions. Although this network includes obvious disease interactions like horizontal and vertical transmission, it also includes ecologic interactions that may be relatively independent of the disease itself. In the LACV system, for example, competition between native tree-hole mosquitoes and invasive tiger mosquitoes strongly influences whether or not LACV persists (Figure 4). Indeed, as a consequence of this competition, tiger mosquitoes can drive local extinction of LACV, despite the fact that tiger mosquitoes can acquire and transmit the virus, making them seem to be competent vectors.

Because of the complexity of disease transmission in ecologic systems, it is often hard to identify the causes of altered disease epidemiology. However, faced with increasing climate and landscape change, ongoing introduction of novel invasive species (pathogens and vectors), and emerging or reemerging diseases, an understanding of the effects of these different forms of global change on disease dynamics is essential. We have moved toward this goal by developing a framework for investigating the role of invasive vectors on the transmission of a native disease. Using LACV as an example, our model highlights the fact that the introduction of a new disease vector does not guarantee increased disease transmission and, in fact, can even drive local extinction of an endemic pathogen. 
